# Characteristic of molecular subtype based on lysosome-associated genes reveals clinical prognosis and immune infiltration of gastric cancer

**DOI:** 10.3389/fonc.2023.1155418

**Published:** 2023-05-01

**Authors:** Maodong Hu, Ruifeng Chong, Weilin Liu, Shuangyong Liu, Xiaolei Liu

**Affiliations:** ^1^ Department of Gastroenterology, Huangdao District People’s Hospital, Qingdao, China; ^2^ Department of General Surgery, Chengyang District People’s Hospital, Qingdao, China; ^3^ General Surgery Department, Qingdao Hongdao People's Hospital, Chengyang District Center for Disease Control and Prevention, Qingdao, China; ^4^ Department of Thyroid and Breast Surgery, The Affiliated Hospital of Qingdao University, Qingdao, China; ^5^ Department of Gastrointestinal Surgery, The Affiliated Hospital of Qingdao University, Qingdao, China

**Keywords:** gastric cancer, lysosome-associated genes, molecular subtype, risk model, prognostic prediction

## Abstract

**Background:**

Lysosome are involved in nutrient sensing, cell signaling, cell death, immune responses and cell metabolism, which play an important role in the initiation and development of multiple tumors. However, the biological function of lysosome in gastric cancer (GC) has not been revealed. Here, we aim to screen lysosome-associated genes and established a corresponding prognostic risk signature for GC, then explore the role and underlying mechanisms.

**Methods:**

The lysosome-associated genes (LYAGs) were obtained from MSigDB database. Differentially expressed lysosome-associated genes (DE-LYAGs) of GC were acquired based on the TCGA database and GEO database. According to expression profiles of DE-LYAGs, we divided the GC patients into different subgroups and then explored tumor microenvironment (TME) landscape and immunotherapy response in LYAG subtypes using GSVA, ESTIMATE and ssGSEA algorithms. Univariate Cox regression analysis, LASSO algorithm and multivariate Cox regression analysis were adopted to identify the prognostic LYAGs and then establish a risk model for patients with GC. The Kaplan-Meier analysis, Cox regression analysis and ROC analysis were utilized to evaluate the performance of the prognostic risk model. Clinical GC specimens were also used to verify the bioinformatics results by qRT-PCR assay.

**Results:**

Thirteen DE-LYAGs were obtained and utilized to distinguish three subtypes in GC samples. Expression profiles of the 13 DE-LYAGs predicted prognosis, tumor-related immunological abnormalities and pathway dysregulation in these three subtypes. Furthermore, we constructed a prognostic risk model for GC based on DEG in the three subtypes. The Kaplan-Meier analysis suggested that higher risk score related to short OS rate. The Cox regression analysis and ROC analysis indicated that risk model had independent and excellent ability in predicting prognosis of GC patients. Mechanistically, a remarkable difference was observed in immune cell infiltration, immunotherapy response, somatic mutation landscape and drug sensitivity. qRT-PCR results showed that compared with corresponding adjacent normal tissues, most screened genes showed significant abnormal expressions and the expression change trends were consistent with the bioinformatics results.

**Conclusions:**

We established a novel signature based on LYAGs which could be served as a prognostic biomarker for GC. Our study might provide new insights into individualized prognostication and precision treatment for GC.

## Introduction

Gastric cancer (GC) is one of the common digestive carcinomas with high incidence rate and mortality. Up to 770 000 people die of GC worldwide each year, Chinese patients account for more than 370,000 (1). Studies have shown that the prognosis of advanced gastric cancer is generally poor. Despite new breakthroughs in surgical techniques, radiotherapy, chemotherapy and targeted therapy, the 5-year survival rate is still less than 50%, while the 5-year survival rate of early patients is more than 90% (2, 3). Therefore, early screening and prognostic prediction of gastric cancer are of great significance to improve the survival of patients.

Lysosomes are membrane-bound intracellular organelles, which are made up of acid lumen and a layer of lysosomal membrane (4, 5). Recent discoveries revealed that lysosomes played a critical role in degradation, innate and adaptive immunity, nutrient sensing and signal pathways (5–7). In addition, the essential role of the lysosome-related mechanisms has been reported in progressions of malignancies (8). Autophagy as the overarching lysosomal activity, can suppress tumorigenesis by inhibiting the transformation of pre-malignant cells, eliminating dysfunctional mitochondria and suppressing genomic instability (9–11). Moreover, lysosome has been demonstrated to contribute to tumor progression *via* regulating tumorigenesis, proliferation, invasion, radiotherapy resistance, and chemotherapy resistance (5). According to these findings, a series of researches targeting lysosome-related mechanisms for tumor therapy are appealing and have shown promising effects, such as choloroquine derivatives, V-ATPase inhibitors, cathepsin inhibitors and other potential lysosomal targeting agents (8, 12, 13).

Lysosome associated membrane protein family members are important participants in lysosome function, and their roles in GC have recently been gradually understood. Lysosome associated membrane protein family member 1 (LAMP1) has been reported to be associated with the risk of gastric cancer caused by Helicobacter pylori (14). Lysosome associated membrane protein family member 5 (LAMP5) was up-regulated in metastatic GC. Knockout of LAMP5 significantly inhibited proliferation, invasion and migration of GC cells, and increased apoptosis and cell cycle arrest (15). The significant association of upregulated LAMP5 with poor prognosis in GC also suggests the potential of using lysosome associated membrane proteins as therapeutic targets. Targeted therapies applying agents that bind to lysosome associated membrane proteins also show an initial effect (16). Despite significant advances in the lysosome field of research have been achieved, the particular biological functions and effect of lysosome in GC have not been clarified.

In this study, we comprehensively analyzed lysosome-associated genes in GC and constructed a corresponding prognostic signature, which can contribute to predict the clinical outcomes of GC and provide novel perspectives into potential new drug candidates and specific antitumor targets for GC.

## Materials and methods

### Data arrangement

The publicly available expression matrix, somatic mutation data and copy number variation (CNV) files of gastric cancer (GC) were downloaded from the TCGA database (https://portal.gdc.cancer.gov/). The matrix file of GSE84337 was acquired from the GEO database (https://www.ncbi.nlm.nih.gov/geo/). The corresponding clinical information of samples was obtained from the TCGA (32 normal samples and 371 GC samples) and GSE84437 database (433 GC samples). The GC samples without survival time were excluded in this study (17, 18). The expression profile of GC samples from the TCGA database were transformed from FPKM to TPM and merged the 2 expression profiles *via* R package “sva”.

### Collection of lysosome-associated genes and differential expression analysis

The LYAGs were acquired from the MSigDB database (https://www.gsea-msigdb.org/), and a total of 163 LYAGs were obtained for the next analysis (**Supplementary Table 1**). With the screening criteria set at |fold change| ≥ 2, and adjust p < 0.05, the differential expression analysis was carried out, and the DE-LYAGs were included for the subsequent analysis. The genetic mutation frequency of DE-LYAGs were explored using R package “maftools” from the TCGA database. The CNV of amplification and deletion for DE-LYAGs was investigated based on the TCGA database. R package “RCircos” was utilized to explore the DE-LYAGs position in chromosomal.

### Consensus clustering analysis of LYAGs

R package “ConsensuClusterPlus” was carried out to identify the molecular subtypes of GC samples based on the expression profile of DE-LYAGs. Firstly, after the 1000 iterations of validation, the GC samples were classified into the optimal molecular subtypes. Second, the R package “survival” was utilized to explore the clinical prognosis of GC in the LYAGs-based molecular subtypes. Principal component analysis (PCA) was used to illustrate the distribution pattern of the unsupervised consensus clustering *via* “ggplot2” package. Finally, R package “pheatmap” was employed to determine the association of clinical features and DE-LYAGs expression profile in the LYAGs-based molecular subtypes. R package “GSVA” was performed to explore the KEGG terms in LYAGs-based subtypes on the basis of “c2.cp.kegg.v7.2.symbols.gmt” reference gene set.

### Identification of LYAG subtype-related differential expression genes (DEGs) and gene cluster generation

To determine the characteristic of biological function of the LYAG-based molecular subtypes in GC, we conducted a differential analysis between the LYAG-based molecular subtypes. R package “limma” was employed to calculate the DEGs between molecular subtypes with the filter criteria set at |fold change| >1 and adjust p < 0.05. Then, the overlapping DEGs between molecular subtypes was included for the next investigation. Gene oncology (GO) and kyoto encyclopedia of genes and genomes (KEGG) algorithms were carried out to explore the biological function and signaling pathways of DEGs *via* R package “clusterProfiler”. On the basis of these DEGs, R package “ConsensuClusterPlus” was utilized to cluster the GC samples into different gene clusters.

### Characteristics of tumor microenvironment

The TME landscape of GC patients was explored using ESTIMATE and single sample gene set enrichment analysis (ssGSEA) algorithms. In first, on the basis of the merged expression profile of GC samples, the ESTIMATE scores of each GC sample were evaluated *via* “estimate” package. Then, according to the marker gene of 23 type immune cells, the immune infiltration level of 23 kind immune cells was calculated *via* “GSVA” package. Finally, the expression of immune checkpoints (ICPs) was explored in the different groups, such as PD-L1, PDCD1, LAG3, and CTLA-4.

### Immunotherapy response and chemotherapeutic drugs prediction

The immunophenoscore (IPS) of GC patients was predicted based on the TCIA database (https://tcia.at/home). As a way to predict response to immunotherapy, IPS was commonly used in predicting the response to PD-1 or CTLA-4 treatment. Based on the genomics of drug sensitivity in cancer (GDSC) database (https://www.cancerrxgene.org/), we further explored the chemotherapeutic drugs sensitivity for GC patients *via* “pRRophetic” package.

### Calculation of the risk score

On the basis of expression profile of DEGs, univariate Cox regression analysis (uniCox) was carried out to identify the prognostic variates. Then, the least absolute shrinkage and selection operator (LASSO) method was used to narrow the range of characteristic variables. Multivariate Cox analysis (multiCox) was conducted to explore the independent prognostic variates from the characteristic variables. The risk score of GC samples was calculated by the following algorithm: risk score = gene expression profile (1) x coefficient (1) + gene expression profile (2) x coefficient (2) +……+ gene expression profile (n) x coefficient (n). According to the independent prognostic variates, the GC samples were divided into training cohort and validation cohort with the division threshold set at 7:3, and calculated the risk score, respectively.

### Nomogram development and independent prognostic analysis

Combined with the clinicopathological characteristics and LYAG score of GC samples, a nomogram was developed to assess the clinical survival probability for GC at 1-, 3-, and 5 years *via* R package “rms”. R package “survival” was utilized to assess the overall survival (OS) rate for GC samples in risk and molecular subgroups. Under the estimation of univariate and multivariate Cox analysis, the independence of LYAG score was explored.

### Real-time quantitative fluorescence PCR

The experiment has been granted approval by both the *Human Ethics Committee of the Affiliated Hospital of Qingdao University and the Ethics Office of Qingdao University*. Samples of tumor and paired adjacent tissues were collected from patients diagnosed with GC. RNA extraction from both types of tissues was carried out using Trizol reagent (Cat# 15596018, Thermo). The cDNA was then synthesized using a RT kit with gDNA Eraser (Perfect Real Time), followed by real-time quantitative qRT-PCR (Cat# RR047A, Takara) for further analysis. Finally, the mRNA expression levels were measured using SYBR Pre-mix Ex Taq II (TliRNaseH Plus) (Cat# RR820B, Takara). PCR primer pairs were attached as **Supplementary Table 2**.

### Statistical analysis

The data preprocessing and analysis were carried out in Perl and R language environment. Statistical algorithms were conducted as follows: Wilcoxon rank-sum test was adopted for statistics in two groups, and ANOVA test was employed for statistics in multiple groups, with p < 0.05 considered statistically different.

## Results

### The differential expression and genetic mutation characteristic of LYAGs in GC

**Figure 1:**
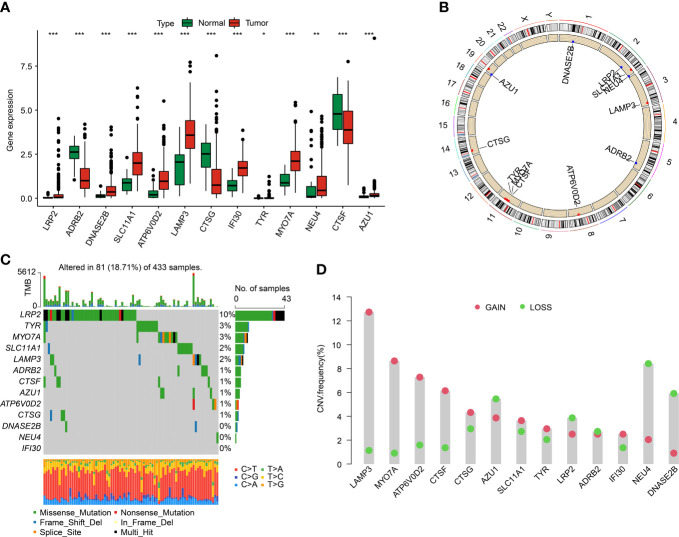
Differential expression analysis and somatic mutation feature of LYRGs in GC. **(A)** The differential expression analysis of LYAGs in normal and tumor tissues from TCGA. **(B)** Position of LYAGs on chromosome. **(C)** Genetic mutation characteristics of LYAGs in GC from GSE84437. **(D)** The CNV of LYAGs. *p < 0.05, **p < 0.01, ***p < 0.001.

We identified 13 DE-LYAGs based on TCGA database to explore the role in tumorigenesis of GC. The differential expression analysis results suggested that the expression level of LRP2, DNASE2B, SLC11A1, ATP6V0D2, LAMP3, IFI30, TYR, MYO7A, NEU4, and AZU1 was overexpressed in tumor tissues, whereas the expression level of ADRB2, CTSG, and CTSF was expressed higher in normal tissues (**Figure 1A**). The location of DE-LYAGs in chromosome was illustrated in **Figure 1B**. Additionally, the somatic mutation characteristic showed a significant mutation frequency of DE-LYAGs in 81 (18.71%) of 433 samples from GSE84437 database, with the mutation frequency of LRP2, TYR, and MYO7A was 10%, 3%, and 3%, respectively (**Figure 1C**). We further explored the CNV of DE-LYAGs in GC, and the result indicated that the LAMP3, MYO7A, ATP6V0D2, CTSF, and CTSG displayed higher amplification; however, the CNV of AZU1, LRP2, NEU4, and DNASE2B displayed higher deletion (**Figure 1D**). These results illustrated the difference of LYAGs in expression level and somatic mutation landscape, showing a potential role of LYAGs in tumorigenesis of GC.

**Figure 2:**
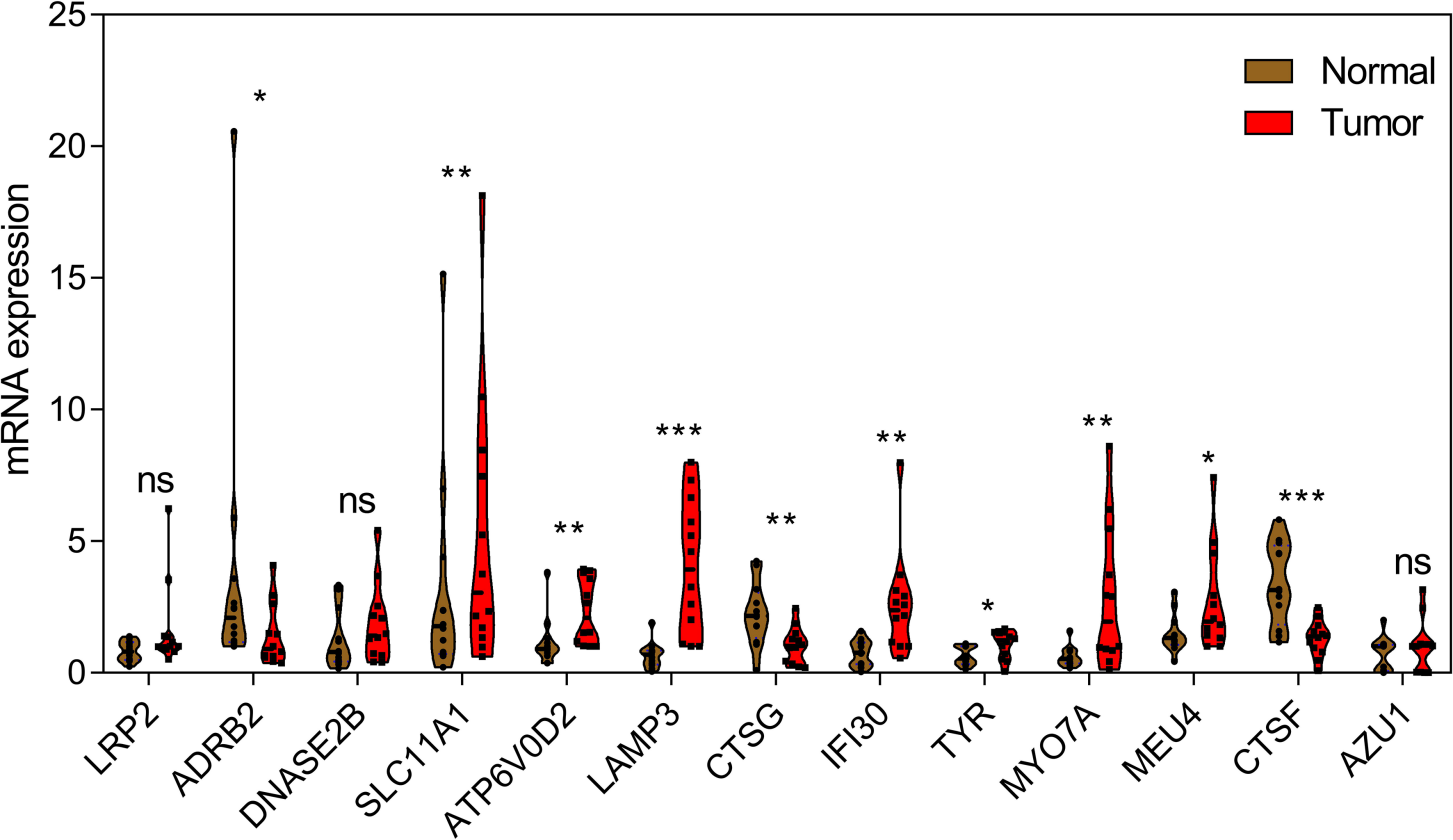
qRT-PCR analysis of 13 LYAGs in clinical samples in GC and corresponding adjacent normal tissues. *p < 0.05, **p < 0.01, ***p < 0.001. ns, no significance.

In order to further verify the abnormal expressions of LYAGs in GC, GC clinical specimens were collected and compared with corresponding adjacent normal tissues by qRT-PCR to detect the mRNA level of the selected LYAGs. qRT-PCR results showed that compared with adjacent normal tissues, among the 13 genes we screened, 10 genes showed significant abnormal expression in the collected case specimens, and the expression change trend was consistent with the bioinformatics results (**Figure 2**).

### Identification of LYAGs subgroups for GC

**Figure 3:**
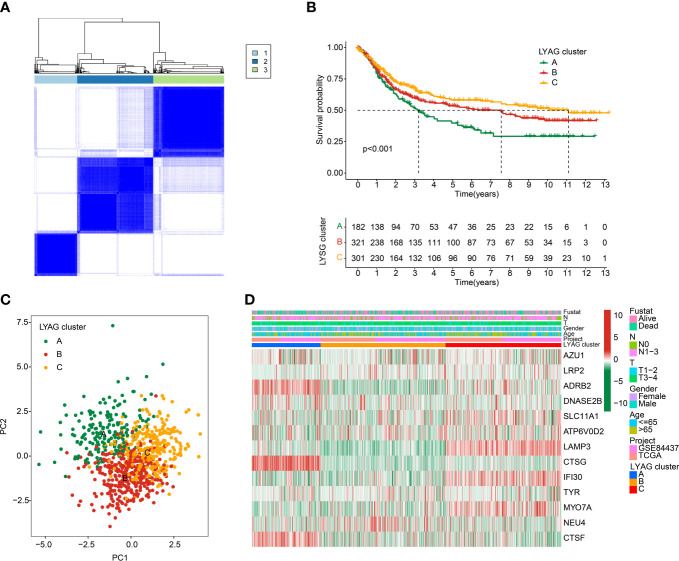
Identification of molecular subtypes for GC. **(A)** Unsupervised consensus clustering analysis for GC. **(B)** Clinical survival outcome of GC in LYAG clusters. **(C)** PCA score plot of LYAG cluster subgroups. **(D)** Characteristics of clinicopathological and LYAGs expression in GC subtypes.

To explore molecular subtypes of LYAGs in the tumorigenesis of GC, we enrolled the data of TCGA-STAD and GSE84437 dataset, and 804 samples were collected for the next investigation. Based on the expression characteristic of 13 LYAGs in GC, an unsupervised consensus clustering algorithm was carried out to identify the molecular subtypes. The heatmap suggested an optimum classification with k = 3, and the 804 GC samples were clustered into LYAG cluster A (n = 182), cluster B (n = 321), and cluster C (n = 301) (**Figure 3A**). The Kaplan-Meier survival analysis showed a clear difference in clinical prognostic outcome of GC, which the OS rate of patients in LYAG cluster A was greatly lower compared to those in LYAG cluster B and C (**Figure 3B**, p < 0.001). PCA plot revealed that the GC samples in LYAG cluster A, B and C could be clearly distinguished (**Figure 3C**). In addition, the clinicopathological characteristics and 13 LYAGs expression were displayed in a heatmap, and the result indicated a significant difference between the LYAG cluster A, B, and C (**Figure 3D**).

### Characteristic of TME and immunotherapy response for GC

**Figure 4:**
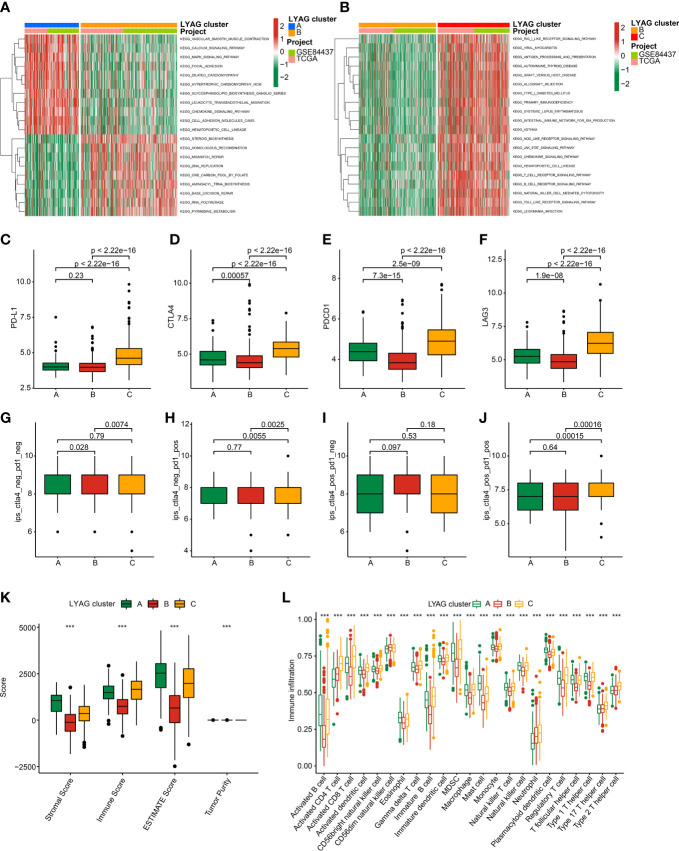
TME landscape and immunotherapy response of GC in LYAG subtypes. **(A, B)** GSVA of KEGG signaling pathways between LYAG subtypes. **(C–F)** Expression profile of PD-L1, CTLA4, LAG3, and PDCD1in LYAG subtypes. **(G–J)** IPS evaluation shows the response to PD-1 and CTLA-4 of GC in LYAG subtypes. **(K)** ESTIMATE assessment. **(L)** Immune infiltration of 23 type immune cells in LYAG cluster A, B and C. ***p < 0.001.

The potential regulation signaling pathways of GC in LYAG subgroups were explored *via* GSVA algorithm. The GSVA results suggested a significant difference of the signaling pathways associated with tumorigenesis and immune regulation between LYAG cluster A and B, such as MAPK signaling pathways and chemokine signaling pathways (**Figure 4A**). Notably, a serial of immune associated pathways was remarkably up-regulated in LYAG cluster C, involving in T cell receptor signaling pathways, B cell receptor signaling pathways, and natural killer cell mediated cytotoxicity (**Figure 4B**). These results indicated that immune associated signaling pathways may play a critical function in the development of GC. The ICPs results suggested the expression of PD-L1, CTLA4, PDCD1, and LAG3 was higher in LYAG cluster C than other LYAG cluster subgroups (**Figures 4C–F**). IPS results showed a clear difference in immunotherapy response of GC samples in different LYAG molecular subtypes. The GC samples in LYAG cluster C showed a better response to PD-1, and PD-1/CTLA4 treatment (**Figures 4G–J**). The score of ESTIMATE assessment displayed a higher stromal, immune and ESTIMATE scores and lower tumor purity of GC in LYAG cluster C (**Figure 4K**). The immune infiltration of 23 type immune cells illustrated a notable difference of GC samples in the LYAG subtypes, such as CD 8+ T cell, CD 4+ T cell, MDSC, mast cell and neutrophil (**Figure 4L**).

### Generation of gene clusters based on LYAG subtypes-related differential expression genes

**Figure 5:**
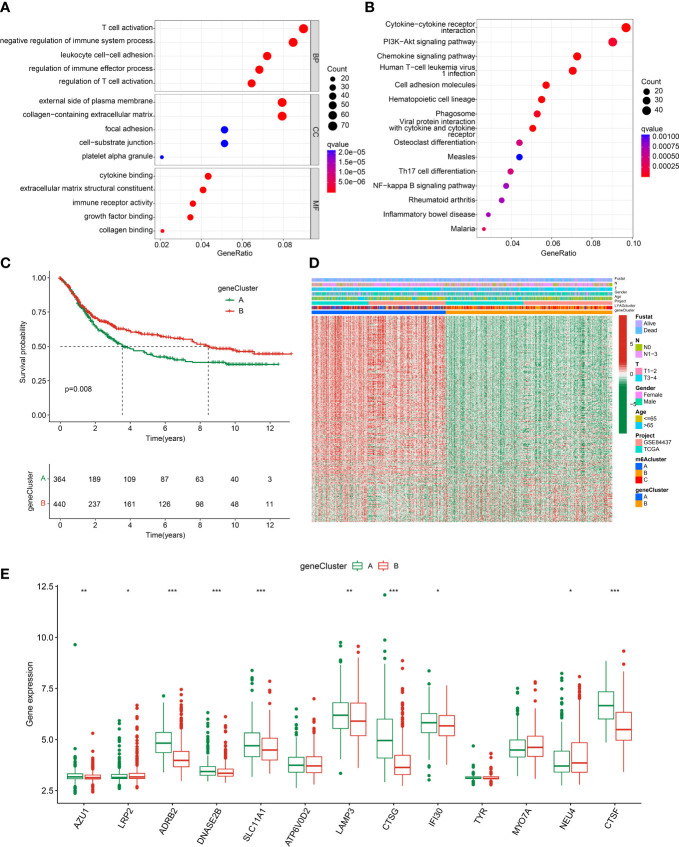
Gene cluster analysis based on the LYAG subtypes-related DEGs. **(A, B)** GO and KEGG enrichment analysis of LYAG subtype-related DEGs. **(C)** Prognostic survival analysis of GC in gene clusters. **(D)** Clinicopathological characteristics and DEGs expression profile of GC in gene clusters. **(E)** LYAGs expression in gene cluster A and B. *p < 0.05, **p < 0.01, ***p < 0.001.

To explore the potential molecular function of LYAG subtypes, we performed a differential expression analysis between LYAG subtypes to identify the DEGs. Under the selection filter set at |fold change| > 1 and p value < 0.001, 840 overlapping DEGs between the LYAG subtypes were obtained (**Supplementary Figure 1**). GO enrichment analysis suggested that the DEGs were linked with T cells activation, negative regulation of immune system process, leukocyte cell−cell adhesion, external side of plasma membrane, and cytokine binding (**Figure 5A**). KEGG revealed that the DEGs were enriched in cytokine−cytokine receptor interaction, PI3K−Akt signaling pathway, and chemokine signaling pathway (**Figure 5B**). Next, an unsupervised consensus clustering was carried out to cluster the GC samples based on the DEGs, and 2 gene clusters (Cluster A: 364, Cluster B: 440) were successfully obtained. The clinical prognostic outcome analysis revealed that the OS rate of GC patients in gene cluster A was worser than those patients in gene cluster B (**Figure 5C**, p = 0.008). The characteristics of clinicopathological and expression profile of DEGs were illustrated in a heatmap, and the result suggested that most of DEGs were greatly lower in gene cluster B (**Figure 5D**). Furthermore, the expression of LYAGs in gene cluster indicated that the expression of AZU1, ADRB2, DNASE2B, SLC11A1, LAMP3, CTSG, IFI30, and CTSF were clearly higher in gene cluster A; however, the expression of LRP2 and NEU4 were higher in gene cluster B (**Figure 5E**).

### Development and validation of the DEGs-based risk model

**Figure 6:**
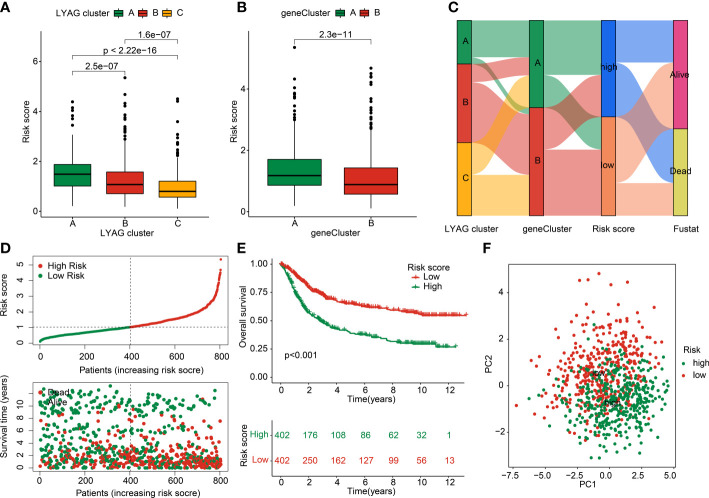
Construction of LYAG subtype-related DEGs risk model in GC. **(A)** Risk score in LYAG subtypes. **(B)** Difference analysis of risk score in gene cluster. **(C)** Relationship of risk score and LYAG cluster, gene cluster and clinical survival status. **(D)** Risk model construction for GC. **(E)** Clinical prognostic analysis of GC samples in low- and high-risk groups. **(F)** PCA plot.

To explore the prognostic value of LYAG subtypes-related DEGs for GC, a risk model was developed to investigate the clinical prognosis outcome. According to the univariate Cox analysis and LASSO algorithm, 24 features prognostic DEGs were obtained. Additionally, 11 prognostic DEGs were selected from the 24 features prognostic DEGs to develop the risk model of GC *via* multivariate Cox analysis. In LYAG cluster subtypes, the GC samples in LYAG cluster C with better clinical prognostic outcome had lower risk score (**Figure 6A**). Meanwhile, the same result was observed of GC samples in gene cluster A and B (**Figure 6B**). The Sankey plot illustrated the relationship between LAYG cluster, gene cluster, risk score and clinical survival status (**Figure 6C**). Based on the DEGs prognostic signature, the GC samples were divided into training cohort (n = 563) and test cohort (n = 241) with the classification radio set at 7: 3. In the entire risk cohort, the GC samples were classified into low- and high-risk groups based on the median risk score, and the results showed that the GC samples with low-risk score had better clinical survival status (**Figure 6D**). Kaplan-Meier analysis of GC samples in low- and high-risk groups suggested that the OS rate of those GC with low-risk score was notably better than those with high-risk score (**Figure 6E**, *p* < 0.001). PCA plot demonstrated that the GC samples in low- and high-risk groups could be clearly distinguished based on the prognostic signature (**Figure 6F**). Of note, the clinical survival outcome of GC samples in the training cohort and test cohort showed a similar result to the entire cohort, for those GC samples in low-risk group, the OS rate was better than in high-risk group (**Supplementary Figure 2**). Overall, these results demonstrated that the risk model of LYAG subtypes-related DEGs could accurately evaluate the clinical outcome of GC and could be considered as independent factor for GC.

### Independent prognostic analysis of risk model in GC

**Figure 7:**
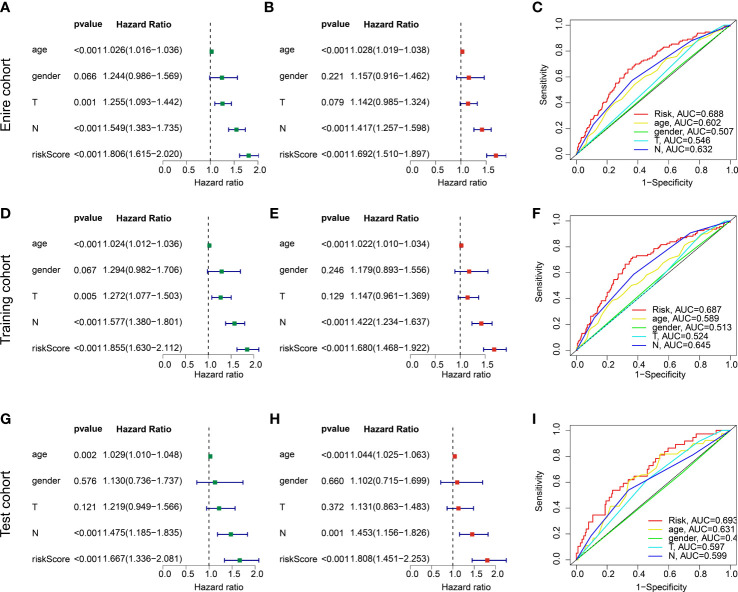
independent prognosis analysis of risk score and predictive ability evaluation. **(A, B)** Univariate and multivariate Cox analysis in entire cohort. **(C)** ROC curve. **(D–F)** Independence analysis and ROC curve in the training cohort. **(G–I)** Independence analysis and ROC curve in the test cohort.

Combined with the clinical characteristics from TCGA and GSE84437, the independence of the risk model was further explored in the entire training and test cohorts. In the entire cohort, univariate Cox analysis displayed that age (HR = 1.026 (1.016-1.036), p < 0.001), T (HR = 1.255(1.093-1.442), p = 0.001), N (HR = 1.549(1.383-1.735), p < 0.001), and risk score (HR = 1.806(1.615-2.020), p < 0.001) were associated with worse clinical prognosis (**Figure 7A**). Multivariate Cox analysis showed that N (HR = 1.417(1.257-1.598), p < 0.001) and risk score (HR = 1.692(1.510-1.897), p < 0.001) were an independent factor for GC (**Figure 7B**). The predictive ability of risk model and clinicopathological characteristics (age, gender, N and T) revealed that the AUC of risk model (0.688) was better than other clinicopathological characteristics (**Figure 7C**). In the training and test cohorts, the independence of risk model and ROC curve results demonstrated that the risk score could be considered as an independent prognostic indicator for GC and showing a good predictive ability than other clinical features (**Figures 7D–I**).

### Development of nomogram of risk score and clinicopathological characteristics in GC

**Figure 8:**
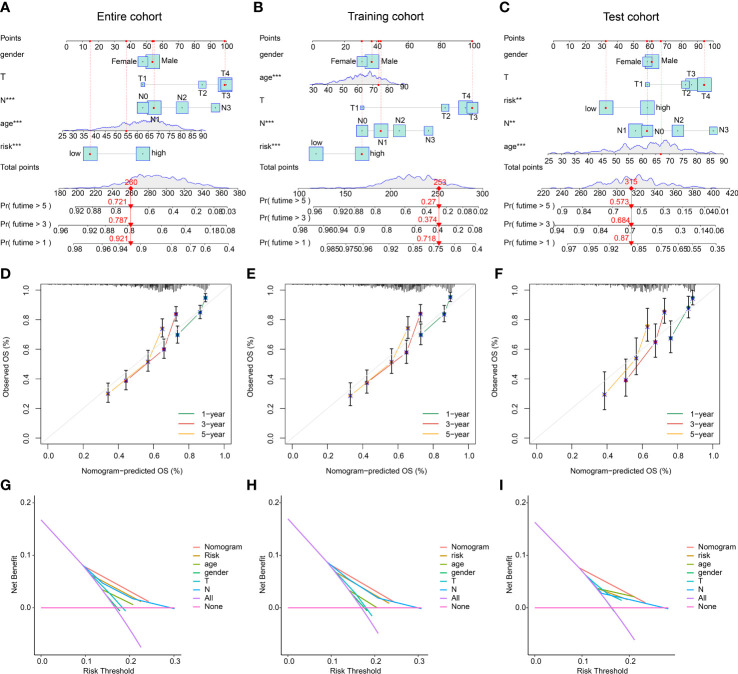
Nomogram development and DCA evaluation. **(A–C)** Nomogram construction of risk score and clinical features in entire, training, and test cohorts. **(D–F)** Calibration curve analysis. **(G–I)** DCA curve of nomogram, risk score and clinical features. **p < 0.01, ***p < 0.001.

Based on the risk score and different clinicopathological characteristics, a nomogram was developed to explore the survival probability of GC in 1-, 3-, and 5-year in entire, training, and test cohorts (**Figures 8A–C**). Calibration curve revealed that the survival probability (1-, 3-, and 5-years) evaluated by nomogram was accurate for GC in entire, training, and test cohorts (**Figures 8D–F**). Additionally, the DCA results in entire, training, and test cohorts showed the predictive ability of nomogram in predicting prognosis for GC was notably better than risk model and clinicopathological characteristics (**Figures 8G–I**). These results demonstrated that the nomogram development of risk score and clinicopathological features to evaluate prognosis of GC patients was compelling and dependable.

### The TME landscape and immunotherapy response of GC in risk subgroups

**Figure 9:**
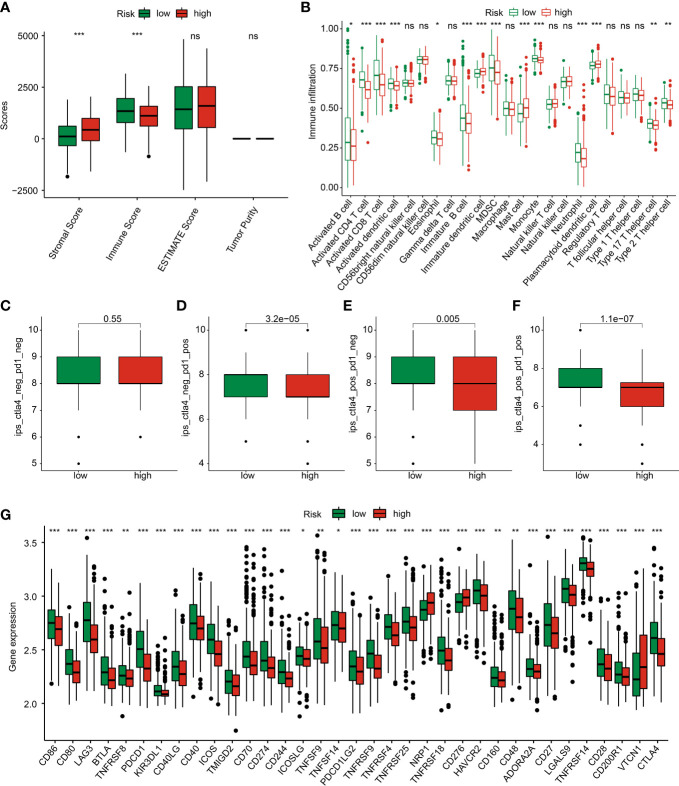
TME landscape and immunotherapy response of GC in the risk subgroups. **(A)** ESTIMATE score. **(B)** The proportion of 23 immune cells estimated by ssGSEA. **(C–F)** IPS score. **(G)** Immune checkpoints expression. *p < 0.05, **p < 0.01, ***p < 0.001. ns, no significance.

Giving the clear enrichment of DEGs in immune-related signaling pathways, the TME landscape and potential immunotherapy response of GC in the risk subgroups were further explored. ESTIMATE results showed that the GC patients with low-risk score had lower stromal score and high immune score (**Figure 9A**). Immune infiltration assessment of 23 immune cells by ssGSEA suggested a clear difference in most immune cells between the low- and high-risk groups, such as activated B cell, CD4 + T cell, CD8 + T cell, and MDSC (**Figure 9B**). Moreover, the IPS results revealed that the GC patients in the low-risk group were more sensitive to the PD-1, CTLA-4, and PD-1/CTLA-4 treatment (**Figures 9C–F**). The expression of ICPs displayed that most of the ICPs were expression higher in the low-risk group, implying a better immunotherapy clinical outcome for those GC patients with low- risk score (**Figure 9G**).

### The correlation of risk score and somatic mutation landscape

**Figure 10:**
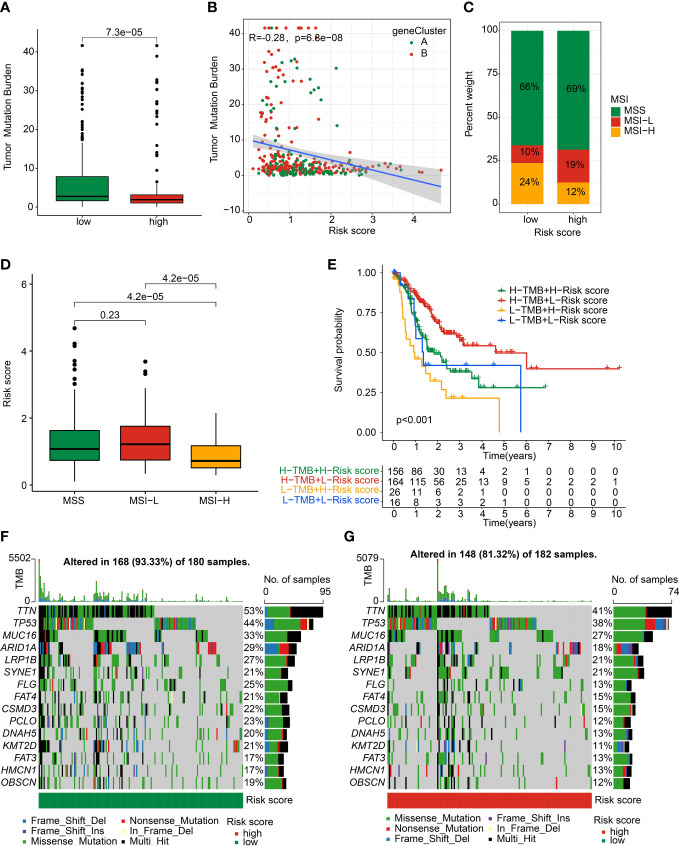
Somatic mutation landscape and MSI in GC. **(A)** TMB analysis. **(B)** Correlation analysis of risk score and TMB in gene cluster. **(C)** Percent of MSI in low- and high-risk groups. **(D)** Distribution of risk score in MSS, MSI-L, and MSI-H. **(E)** Kaplan-Meier analysis of GC with low- and high-risk scores in L- and H-TMB groups. **(F, G)** The genetic mutation frequency in low- and high-risk groups.

The relationship between risk score and TMB was further explored in GC patients. The result showed a higher TMB level in low-risk group of GC patients than high-risk group, and the correlation analysis displayed that the risk score was negatively linked with gene cluster (**Figures 10A, B**). MSI result suggested that the high-risk group had higher percent of MSI-L and lower percent of MSI-H (**Figure 10C**). Notably, the risk score of GC patients with MSI-H was significantly lower than those with MSS and MSI-L (**Figure 10D**). The Kaplan-Meier analysis showed that the clinical prognosis of GC patients with low-risk score was better than those with high-risk score in the low- and high-TMB groups (**Figure 10E**). The genetic mutation landscape illustrated a significant somatic mutation frequency of GC in low- and high-risk groups. As shown in **Figures 10F, G**, 168 (93.33%) of 180 samples were observed somatic mutation in low-risk group, and 148 (81.32%) of 180 samples were observed somatic mutation in high-risk group. The mutation frequency of TTN, TP53, MUC16, ARID1A, and LRP1B was 53%, 44%, 33%, 29% and 27% in low-risk group, which exhibited higher TMB level than in high-risk group.

### Chemotherapy drug prediction in risk subgroups

**Figure 11:**
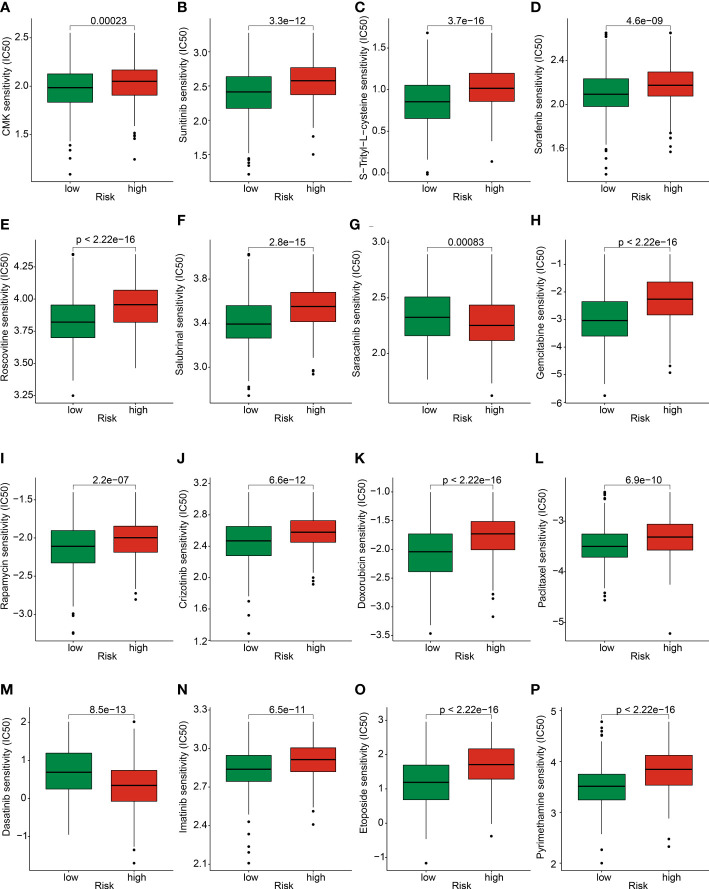
Prediction of chemotherapy drug for GC in risk subgroups. The IC50 distribution of **(A)** CMK, **(B)** Sunitinib, **(C)** S-Trityl-L-cysteine, **(D)** Sorafenib, **(E)** Roscovitine, **(F)** Salubrinal, **(G)** Saracatinib, **(H)** Gemcitabine, **(I)** Rapamycin, **(J)** Crizotinib, **(K)** Doxorubicin, **(L)** Paclitaxel, **(M)** Dasatinib, **(N)** Imatinib, **(O)** Etoposide, and **(P)** Pyrimethamine between low- and high-risk groups.

We further explored several potential chemotherapy drugs which may benefit for the treatment of GC patients in the low- and high-risk groups. Based on the GDSC database, the IC50 of antitumor were predicted and the results suggested that the GC patients with high-risk score were more sensitive to CMK, sunitinib, S-Trityl-L-cysteine, sorafenib, roscovitine, salubrinal, gemcitabine, rapamycin, crizotinib, doxorubicin, paclitaxel, imatinib, etoposide, and pyrimethamine; in addition, the GC patients with low-risk score were more sensitive to saracatinib and dasatinib (**Figure 11**). Overall, these results demonstrated the potential association between the risk model and chemotherapy drugs, providing a new treatment strategy for the drug treatment of GC patients in different risk subgroups.

## Discussion

Despite great progress has been made in diagnosing and treating GC, the improvement of clinical outcome for GC remains unsatisfactory (19). There is still an urgent need to improve survival time for patients with GC. Treatments targeting lysosome have shown promising effects in tumor therapy. Additionally, therapeutic outcomes can be significantly improved when lysosomes-related methods are combined with other conventional therapies (20). Regrettably, the lysosomes in GC and its underlying effect remains largely unknown. Here, according to the expression level of 13 LYAGs in GC samples, we identified three robustly distinct lysosome-related molecular subtypes, Cluster A, Cluster B and Cluster C. These LYAG subtypes had significant differences in prognosis, clinicopathological characteristics, immune infiltration, immune response and functional pathways. The GSVA results revealed that a serial of immune-associated pathways was up-regulated in cluster C. Moreover, immune infiltration results also showed better immunotherapy response, higher immunoreactivity and lower immunosuppression in cluster C. Aggregating all of these findings, we hypothesized that GC patients in different LYAG subtypes would have different prognosis due to different immune response and immune status tumor microenvironment, and these differences contribute to tumor progression. The functional enrichment based on DEGs in LYAG subtypes showed that immune-linked biological processes and pathways were highly enriched, revealing the potential regulatory role of immune-related signaling pathway in the mechanisms of lysosome in patients with GC. Furthermore, derived from LYAG subtypes-related DEGs, we identified 11 prognostic genes and constructed a prognostic signature for GC. Involved immune infiltration, TME landscape and chemotherapy response analysis were then explored.

Conventional therapies, such as surgery, chemotherapy and radiotherapy, have limited clinical efficacy to treat GC, especially advanced-stage gastric cancer (21). Tumor immunotherapy is a new type therapeutic tool for the treatment of various, which has provided a promising avenue to gastric cancer patients, and made a breakthrough in both research area to clinical practice (21–23). Additionally, GC may be an ideal candidate for immunotherapy due to the high incidence of somatic mutations (24). Results from ssGSEA showed patients in the low-risk group had significantly more B cells, CD4/CD8+ T cell and MDSC infiltrating the tumor. B cell can act as a tumor-suppressive factors through producing antitumor-antibody in GC (25). An increase in the number of CD4/CD8+ T cell is correlated with better clinical outcome and well-differentiated in GC(26). It is suggested that GC patients in low-risk were more sensitive to immunotherapy. Moreover, the IPS results revealed that the PD-1, CTLA-4, and PD-1/CTLA-4 treatment is more beneficial to GC patients with low risk. Immunotherapy related to ICIs is a promising method to treat GC (27), however, available clinical results for ICIs display limited clinical impact of monotherapy. Combing the ICIs with targeted therapy, chemotherapy, or other traditional therapies all show a significant improvement in clinical outcome for GC (28). Compared to chemotherapy alone, the anti-PD-1 antibody nivolumab in combination with chemotherapy generates superior clinical efficacy for patients with advanced GC (29). Based on the results of this clinical study, the combinatorial treatment of nivolumab and chemotherapy was approved for patients with advanced or metastatic GC by FDA.

After GSVA clustering enrichment analysis, we observed a significant difference among cluster A, B and C. Cluster C had active enrichment of immune-related pathways, including antigen processing and presentation, TCR signaling and BCR signaling pathways. In the follow-up risk assessment, most patients in group C had a relatively good prognosis. This result suggests the important role of immune response and immunotherapy in STAD. In contrast, cluster A patients had a poor prognosis. The observed enriched MAPK and calcium signaling pathway could be one of the causes (30, 31).

Numerous studies have shown patients with high TMB might be more effective for immunotherapy patients with high TMB due to relatively high number of neoantigens (32–35). In our study, patients’ mutation data showed TMB level was negatively correlated with risk, implying a better clinical outcome of immunotherapy for GC patients in low- risk group. We also found that the top 3 most significantly mutated genes were TTN, TP53 and MUC16, which exhibited higher mutation frequency in low-risk group than high-risk group. TTN is the longest-known gene, which is associated with increased TMB and correlated with objective response to immune checkpoint blockade in solid tumors (36). Our conclusions showed that the proportion of TTN mutations in high-risk patients was 12% lower than that of low-risk patients, which might lead to differences in ICI treatment reactance. TP53 is the most extensively studied human suppressor gene, when it occurs mutations, not only the tumor suppressive functions are abolished, but also the protein with new pro-oncogenic functions is equipped (37). As for MUC16, is one of the most commonly mutated gene in many human tumors, which is linked with enhanced growth and metastasis capacity of tumor cells (38). Furthermore, MUC16 mutations are correlated with prognosis and cell cycle pathways, predicted tumor mutation and immune response in GC, which may offer guidance to immunotherapy for GC(39). In addition, we found a significantly lower proportion of ARID1A mutations in high-risk patients. ARID1A belongs to a class of chromatin regulatory proteins that function by maintaining access to most promoters and enhancers, thereby regulating gene expression (40). ARID1A mutations usually result in loss of ARID1A expression (41, 42). Combined with the evidence of the tumor suppressive effect of ARID1A in gastric cancer, the poor prognosis of high-risk patients may be related to the deletion of expression caused by ARID1A mutation (43). FLG also had a lower proportion of mutations in the high-risk group. It has been reported that the FLG mutant group showed a higher mutation load and immunogenicity in gastric cancer, resulting in increased sensitivity of gastric cancer to 24 chemotherapy agents (44). Our data also showed an association between a higher proportion of FLG mutations and a better response to STAD therapy.

In conclusion, we identified three lysosome-related molecular subtypes in GC. These subtypes exhibited a significant gap in prognosis, clinicopathological characteristics, immune infiltration, immune response and functional pathways. Furthermore, we established a new signature based on 11 prognostic LYAGs with reliable and independent prognostic ability. We also preliminarily demonstrated the guiding significance of the model for chemoresistance and immunotherapy. Our findings will increase the knowledge of lysosome and provide new perspectives and direction for precise therapeutic intervention in GC.

## Data availability statement

The original contributions presented in the study are included in the article/**Supplementary Material**. Further inquiries can be directed to the corresponding author.

## Author contributions

XL conceived and designed the study. MH contributed the data collection and data analysis. RC conceived the original ideas and composed this manuscript. WL and SL contributed the table and figures of this manuscript. All authors contributed to the article and approved the submitted version.
