# Supplementary material for: Characteristic of molecular subtype based on lysosome-associated genes reveals clinical prognosis and immune infiltration of gastric cancer
Source: Front Oncol. 2023 May 1;13:1155418. doi: 10.3389/fonc.2023.1155418 (PMC10183605; doi:10.3389/fonc.2023.1155418)
Supplement: Supplementary file 1 [file Table_1.docx]

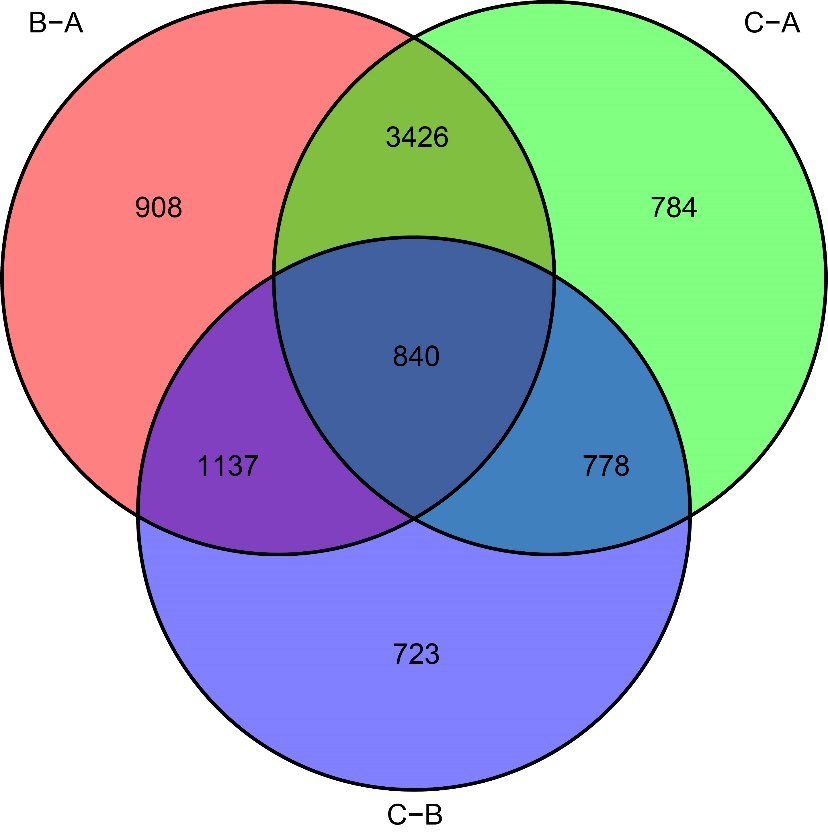


Supplementary Figure 1. Venn shows the overlapping genes between the LYAG molecular subgroups.


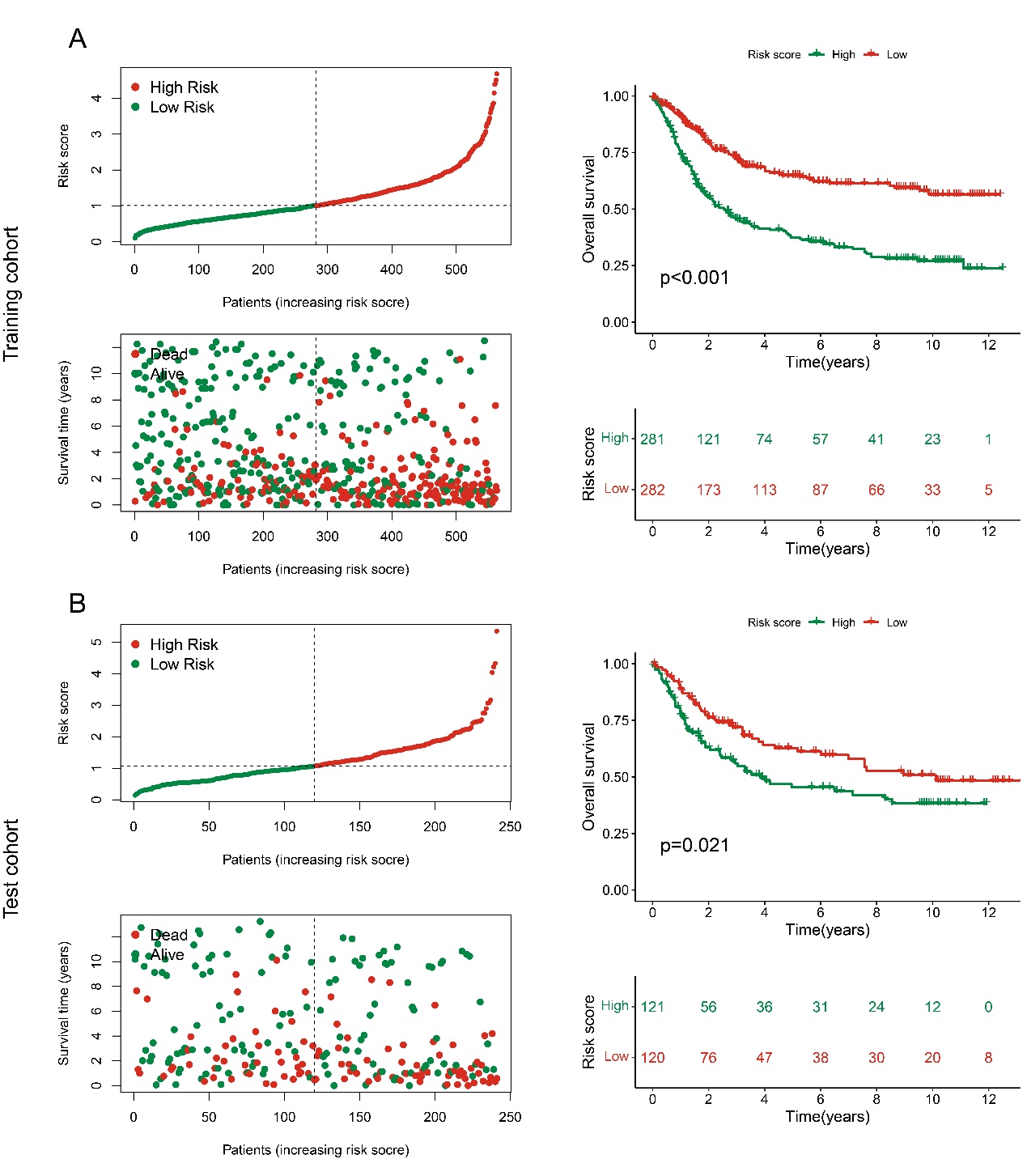


Supplementary Figure 2. Risk model validation in the training cohort (A) and test cohort (B).
